# Supplementary material for: Prevalence and change in alcohol consumption in older adults over time, assessed with self-report and Phosphatidylethanol 16:0/18:1 —The HUNT Study
Source: PLoS One. 2024 May 31;19(5):e0304714. doi: 10.1371/journal.pone.0304714 (PMC11142565; doi:10.1371/journal.pone.0304714)
Supplement: S1 Table — (DOCX) [file pone.0304714.s001.docx]

**S1 Table. Number of participants and participation rate in HUNT2 (1995-97), HUNT3 (2006-08), and HUNT4 (2017-19) surveys, by gender and age groups.**

|  |  | **Women** |  |  | **Men** |  |  | **Total** |  |
| --- | --- | --- | --- | --- | --- | --- | --- | --- | --- |
|  | Invited | Participated | Participation rate | Invited | Participated | Participation rate | Invited | Participated | Participation rate |
| **HUNT2^a^** | N | N | % | N | N | % | N | N | % |
| Age group |  |  |  |  |  |  |  |  |  |
| 60-69 | 5,443 | 4,723 | 87.0 | 5,212 | 4,366 | 84.3 | 10,655 | 9,089 | 85.6 |
| 70-79 | 5,707 | 4,534 | 79.9 | 4,803 | 3,776 | 79.9 | 10,510 | 8,310 | 79.9 |
| 80-89 | 3,338 | 1,960 | 64.7 | 2,075 | 1,242 | 68.2 | 5,413 | 3,202 | 66.0 |
| 90+ | 618 | 266 | 52.8 | 288 | 121 | 52.6 | 906 | 387 | 52.9 |
|  |  | **Women** |  |  | **Men** |  |  | **Total** |  |
| **HUNT3^b^** | N | N | % | N | N | % | N | N | % |
| Age group |  |  |  |  |  |  |  |  |  |
| 60-69 | 6,892 | 5,137 | 74.5 | 6,909 | 4,674 | 67.7 | 13,801 | 9,811 | 71.1 |
| 70-79 | 4,583 | 3,080 | 67.2 | 4,011 | 2,664 | 66.4 | 8,594 | 5,744 | 66.8 |
| 80-89 | 3,394 | 1,335 | 39.3 | 2,102 | 952 | 45.3 | 5,496 | 2,287 | 41.6 |
| 90+ | 662 | 104 | 15.7 | 228 | 49 | 21.5 | 890 | 153 | 17.2 |
|  |  | **Women** |  |  | **Men** |  |  | **Total** |  |
| **HUNT4^c^** | N | N | % | N | N | % | N | N | % |
| Age group |  |  |  |  |  |  |  |  |  |
| 60-69 | 8,219 | 5,745 | 69.9 | 8,511 | 5,430 | 63.8 | 16,730 | 11,175 | 66.8 |
| 70-79 | 6,385 | 4,329 | 67.8 | 6,092 | 3,978 | 65.3 | 12,477 | 8,307 | 66.6 |
| 80-89 | 3,189 | 1,725 | 54.1 | 2,349 | 1,327 | 56.5 | 5,538 | 3,052 | 55.1 |
| 90+ | 954 | 358 | 37.5 | 442 | 185 | 41.9 | 1,396 | 543 | 38.9 |

Abbreviation: HUNT = Trøndelag Health Study

^a^Reference: Holmen et al. 2003 [1]

^b^Reference: Krokstad et al. 2013 [2]

^c^Reference: Åsvold et al. 2022 [3]

**References:**

1. Holmen J, Midtfjell K, Krûger Ø, Langhammer A, Holmen T, Bratberg G, et al. The Nord-Trøndelag Health Study 1995-97 (HUNT 2): Objectives, contents, methods, and participation. Norsk Epidemiologi. 2003;13(1):19-32.

2. Krokstad S, Langhammer A, Hveem K, Holmen TL, Midthjell K, Stene TR, et al. Cohort Profile: the HUNT Study, Norway. International journal of epidemiology. 2013;42(4):968-77. Epub 20120809. doi: 10.1093/ije/dys095. PubMed PMID: 22879362.

3. Åsvold BO, Langhammer A, Rehn TA, Kjelvik G, Grøntvedt TV, Sørgjerd EP, et al. Cohort Profile Update: The HUNT Study, Norway. International journal of epidemiology. 2023;52(1):e80-e91. doi: 10.1093/ije/dyac095. PubMed PMID: 35578897; PubMed Central PMCID: PMCPMC9908054.
