# Supplementary material for: Prevalence and change in alcohol consumption in older adults over time, assessed with self-report and Phosphatidylethanol 16:0/18:1 —The HUNT Study
Source: PLoS One. 2024 May 31;19(5):e0304714. doi: 10.1371/journal.pone.0304714 (PMC11142565; doi:10.1371/journal.pone.0304714)
Supplement: S2 Table — (DOCX) [file pone.0304714.s002.docx]

**S2 Table. Comparison of participants (≥65 years) with and without measured PEth at HUNT3 (2006-08)**

|  | PEth No | PEth Yes | p-value |
| --- | --- | --- | --- |
| ***HUNT3*** | | | |
| N Age  n  mean (SD) Gender, female  n/N (%) After-tax income (NOK)^a^  n  mean (SD) Urban/rural living, urban  n/N (%) Civil status, living with spouse/partner  n/N (%) Smoking  Never smoked, n/N (%)  Former smoker, n/N (%)  Smoker, n/N (%) | 5,835  5,835 74.2 (6.6)  3,230/5,835 (55.4)  5,831 196,714 (99,199)    3,284/5,777 (56.8)      3,591/5,827 (61.6)    2,323/5,514 (42.1)  2,265/5,514 (41.1) 926/5,514 (16.8) | 6,068  6,068 73.6 (6.2)  3,158/6,068 (52.0)  6,068  190,677 (104,282)  3,920/6,000 (65.3)      3,841/6,067 (63.3)  2,317/5,793 (40.0) 2,495/5,793 (43.1) 981/5,793 (16.9) | <0.001^1^    <0.001^2^    0.001^1^    <0.001^2^      0.058^2^    0.054^2^ |

^1^ Independent-samples t-test; ^2^ χ^2^-test

Abbreviations: HUNT = Trøndelag Health Study; n/N = number; NOK = Norwegian kroner; PEth = Phosphatidylethanol 16:0/18:1; SD = Standard Deviation

^a^ Income after taxes (NOK, 2006-08), values of 0- or negative income for the year of participation were replaced by average of the remaining two values (or one value if only single value available), 0-income for all three years were replaced with missing.
