# Supplementary material for: Prevalence and change in alcohol consumption in older adults over time, assessed with self-report and Phosphatidylethanol 16:0/18:1 —The HUNT Study
Source: PLoS One. 2024 May 31;19(5):e0304714. doi: 10.1371/journal.pone.0304714 (PMC11142565; doi:10.1371/journal.pone.0304714)
Supplement: S3 Table — (DOCX) [file pone.0304714.s003.docx]

**S3 Table. Comparison of participants (≥65 years) with and without measured PEth at HUNT4 (2017-19)**

|  | PEth No | PEth Yes | p-value |
| --- | --- | --- | --- |
| ***HUNT4*** | | | |
| N Age  n  mean (SD) Gender, female  n/N (%)  After-tax income (NOK)^a^  n  mean (SD)  Urban/rural living, urban  n/N (%) Civil status, living with spouse/partner  n/N (%) Smoking  Never smoked, n/N (%)  Former smoker, n/N (%)  Smoker, n/N (%) | 9,834    9,834 74.1 (6.8)    5,171/9,834 (52.6)    9,823  306,749 (211,840)  7,405/9,833 (75.3)    6,350/9,830 (64.6)    3,511/9,727 (36.1) 5,391/9,727 (55.4) 825/9,727 (8.5) | 7,290    7,290 74.1 (6.7)    3,829/7,290 (52.5)    7,289  309,764 (228,600)    3,529/7,283 (48.5)      4,688/7,278 (64.4)    2,605/7,237 (36.0)  3,995/7,237 (55.2) 637/7,237 (8.8) | 0.579^1^  0.939^2^    0.373^1^    <0.001^2^      0.803^2^    0.762^2^ |

^1^ Independent-samples t-test; ^2^ χ^2^-test

Abbreviations: HUNT = Trøndelag Health Study; n/N = number; NOK = Norwegian kroner; PEth = Phosphatidylethanol 16:0/18:1; SD = Standard Deviation

^a^ Income after taxes (NOK, 2017-19), values of 0- or negative income for the year of participation were replaced by average of the remaining two values (or one value if only single value available), 0-income for all three years were replaced with missing.
