# Supplementary material for: Prevalence and change in alcohol consumption in older adults over time, assessed with self-report and Phosphatidylethanol 16:0/18:1 —The HUNT Study
Source: PLoS One. 2024 May 31;19(5):e0304714. doi: 10.1371/journal.pone.0304714 (PMC11142565; doi:10.1371/journal.pone.0304714)
Supplement: Attachment — Submitted filename: Response to PLOS ONE.doc [file pone.0304714.s004.doc]

**Vestfold Hospital Trust	The Norwegian National Centre for**

**Ageing and Health**


The Editor in Chief

Emily Chenette	November, 2023

Dear Dr Chenette

Enclosed please find a copy of the manuscript *“**Prevalence and change in alcohol consumption in older adults over time, assessed with self-report and Phosphatidylethanol 16:0/18:1 – The HUNT Study”.* Response to the letter and changes included in the manuscript are written below in red.

Sincerely yours,

## Kjerstin Tevik Ragnhild Bergene Skråstad Jūratė Šaltytė Benth Geir Selbæk

## Sverre Bergh Rannveig Sakshaug Eldholm Steinar Krokstad Anne-Sofie Helvik

Corresponding author

Kjerstin Tevik

Holemslykkjvegen 63

7224 Melhus, Norway

E-mail: kjerstin.e.tevik@ntnu.no

PONE-D-23-36609

Prevalence and change in alcohol consumption in older adults over time, assessed with self-report and Phosphatidylethanol 16:0/18:1 – The HUNT Study Dr. Kjerstin Tevik

1. Please complete all items on the Human Participants Research Checklist that are relevant for your submission, by following this link: <http://journals.plos.org/plosone/s/file?id=dc11/PLOS> Human Participants Research Checklist.docx (Contact us at [plosone@plos.org](mailto:plosone@plos.org) if you cannot access the document.) There may be overlap between the checklist items and other queries listed below; please address any duplicated queries both in your response email and on the checklist itself. Upload the completed Human Participants Research Checklist as file type “Other” when you re-submit your manuscript. This document is for internal journal use only and will not be published if your article is accepted. The requested information will help us to assess whether your submission complies with PLOS ONE’s policies and adheres to applicable reporting standards. Note that your manuscript may be rejected if you provide incomplete or inadequate responses to the checklist questions and that changing the ‘Section/Category’ of your article does not affect this requirement.

Thank you for making us aware of what is missing in our manuscript. The Human Participants Research Checklist is uploaded under file type “Other”. The original ethics approval and an English translation are also uploaded. The recruitment period for individuals who participated in HUNT2, HUNT3 and HUNT4 is added to the method section from line 233:

“The recruitment period for individuals who participated in HUNT2 was from August 15, 1995 to June 18, 1997; in HUNT3 from October 3, 2006 to June 25, 2008; and in HUNT4 from May 5, 2017 to February 21, 2019”.
